# Medical Evidence on the Duration of Human Pregnancy

**Published:** 1826-07

**Authors:** 


### ON THE DURATION OF HUMAN PREGNANCY.*

The trial which gave occasion to the present publication, excited considerable sensation in the world, and was a fertile source of gossip among both maids and matrons, for at least a fortnight, which is a much longer space of time than most wonders occupy in the present æra of human affairs. The medical witnesses, as usual, came in for their share of ridicule, and some of them called forth pretty general and sharp remark; but upon the whole, *they* ought henceforth to stand well with the ladies, for certainly their evidence, upon the present occasion, did not tend to curtail, but rather extend the *privileges* of that important class of society. We have always understood, that the wives of soldiers and sailors were allowed a greater latitude in respect to the duration of pregnancy than other females; but it appears that the decision, in the present instance, and the weight of medical testimony, have extended that prerogative to women in general, high and low, rich and poor.

As the investigation under review involves many important considerations, with respect to medical science and legislation, we think it will not be uninteresting or useless to put upon record, in the pages of this Journal, a concise statement of the case, and a brief view of the evidence given by the principal medical men cited before the august tribunal of the House of Lords.

In this record, we shall keep to the medico-legal question, and not advert to the history of the parties, which was amply detailed in the newspapers. There were two questions involved—what was the longest, and what was the shortest period of utero-gestation? That is, could it extend to 311, or at least 304 days?—and could a child, born two or three days short of five calendar months from conception, grow up to manhood. It is with the *former* of these two questions that we have to do.

---

* Medical Evidence relative to the Duration of Human Pregnancy, as given in the Gardner Peerage Cause, before the Committee for Privileges of the House of Lords, in 1825-6. With Introductory Remarks and Notes. By ROBERT LYALL, M.D. F.L.S. &c. &c. &c. 8vo. Sewed, pp. 104. Burgess and Hill, April, 1826.

Some of the principal accoucheurs of this metropolis were summoned to give evidence, besides a few of inferior note.

"Of the seventeen medical gentlemen examined, five supported the opinion, that the period of human utero-gestation was limited to about nine calendar months, from thirty-nine to forty-weeks, or from 273 to 280 days; or, if we strictly take them at their words, from 270 to 280 days; one of the witnesses, indeed, said from 265 to 280 days. These gentlemen of course gave their negative to the possibility, unless by miracle, that Henry Fenton Jadis, alias Gardner, could have been the product of a 311 days' gestation.

"On the other side, of twelve medical gentlemen, who seemed to agree with respect to the above-mentioned period as the natural time of gestation; most of them maintained the *possibility* that pregnancy might be protracted to nine and a half, ten, or eleven calendar months, *and of course to* 311 *days, the alleged term of gestation*, at which the counter-claimant was born; and thus admitted the possibility that Mr. Henry Fenton Jadis, alias Gardner, might be a ten and a half months' child: and they adduced a variety of cases, with a view of showing that their doctrine was founded on facts." viii.

The calculations respecting the duration of pregnancy have been founded on several circumstances, but there are only two which we deem it necessary to notice—the cessation of the catamenia, and the period of quickening. As for certain sensations supposed to be felt at the instant of conception, we deem them totally unworthy of consideration. The circumstance of a single cohabitation would be the surest evidence no doubt, were there a sufficient number of instances, and these free from any suspicion of fallacy—but this is never likely to be the case.

*Cessation of the Catamenia.*—It is remarked by Dr. Lyall, that "the most usual way of calculating the time of pregnancy, both among practitioners, and by females themselves, is from the time of the disappearance of the catamenia." This, we believe, is not the case—particularly among practitioners. The more general, indeed the only practical way, is to calculate from the middle of the broken period—that is, from ten days or a fortnight after the last appearance. This, in an average number, would be certain to come the nearest to truth. The more intelligent females calculate in this way—and all practitioners, we should imagine, would interrogate their patients on this principle. When females have unusually long or short intervals between the catamenia, it will, of course, alter the case, and must be allowed for accordingly. In respect to the question, can the catamenia continue after pregnancy? we have the authority of Denman, Hamilton, Burns, and others in the decided negative; while Haller, Heberden, Capuron, Francis, and several men of eminence have decided in the affirmative. It is highly probable, that menstruation during pregnancy is a very rare occurrence, and that the great majority of supposed instances are singular coincidences of hæmorrhage, either from the uterus or vagina. Dr. Lyall avers, that bloody discharges never take place during pregnancy, among the rude female peasants of Russia, except from accident. He also suspects that, in a state of barbarism, at least in a cold climate, women rarely become pregnant while suckling.

*Quickening.* It is needless to advert to the ridiculous doctrine, on which some of our laws are founded, that the fœtus acquires a new existence, or in fact begins to live at the period of quickening. Neither need we advert to the various explanations which have been given of the peculiar sensation of quickening—none of which appear satisfactory. All we know is this—that at a certain period the mother feels the motion of the child, and that the first time she feels this, she has generally an accompanying sense of sickness, faintness, or other sensation that particularly arrests her attention, and leads her to record the event. The more important consideration is, what is the time of quickening? Denman says it happens from the tenth to the twelfth week after conception—but most commonly about the sixteenth. Puzos avers, that it sometimes occurs at the end of two months, but most usually at the expiration of eighteen weeks.

"From the combined accounts of ancient and modern writers, and the evidence of a number of the witnesses who were examined in the Gardner Peerage Cause, it appears, first, that *quickening* takes place, *in different individuals,* from the tenth to the twenty-sixth week; and, secondly, that the *period of quickening* is pretty regular *in the same individual; i. e.* if a woman has quickened at the tenth, twelfth, fourteenth, or sixteenth week, with her first child, *cæteris paribus,* she will continue to quicken about the same advancement in all her subsequent pregnancies." xv.

From what has been adduced, it is evident that the period of quickening can assist us but little in calculating the duration of pregnancy, since, at the very most, it can only apply to the individual case, and not to the quickening of other females. It is even very questionable whether the same individual always quickens at nearly the same period.

In respect to the *size of the abdomen,* it is quite absurd to attach any importance to this mark of pregnancy.

*Examination per vaginam.* Some practitioners (see No. 9 of this Journal, page 212) attach considerable importance to examination *per vaginam,* as a means of ascertaining the exact period of pregnancy. We have already expressed our opinion on this subject—an opinion which appears to coincide with that of Dr. Lyall. "An examination in the earliest months of pregnancy can give but little conclusive information; and after quickening, or after the fourth month, it may inform us that a female is some months pregnant: in the after months of gestation it may assist our judgment considerably, but in no case can it alone indicate the *precise period* of pregnancy." xviii.

Our author makes some pertinent remarks on Dr. Collins' case, as reported in our contemporary of the north, and entertains, with us, much doubt as to the patient having gone eleven months in a state of utero-gestation. We agree with Dr. Lyall on another point—that the stethoscope promises but little aid in ascertaining the point under investigation.

The question, as to the duration of pregnancy, is certainly one of great importance, since it may involve the honour and happiness of families, the legitimacy of offspring, and the succession of property. It has been agitated for hundreds of years, and unfortunately, like many other physiological questions, it remains to this hour in an unsettled state. According to the common consent of mankind the mean term of pregnancy is nine calendar months, or about 40 weeks; at the end of which, labour comes on—not on account of any physiological cause which we can discover, but because it is the *command of God,* as was stated by a physiologist in the days of Avicenna. But, although Nature, or Nature's God, has fixed this period, as the mean duration of gestation, in the same way that the days of man are limited to three score and ten years, yet we see no reason in theory, why the period should not be transgressed in the one case as well as in the other. We know indeed that Nature is peculiarly tenacious of uniformity and accuracy in every thing that respects the *propagation* of the species; but still she is not infallible in her objects, nor invariable in the means of attaining them. In admitting this, we are perfectly convinced that nine in ten of the reported cases of protracted pregnancy would, if well sifted, be found to bear the impress (as Dr. Beck expresses it) of vice or error. The evidence adduced in the Gardner cause, however, has tended forcibly to shew us that Nature will not be limited by the opinions of man—that she will not recognize human laws—that she often delights in secrecy, and indulges in whims and aberrations—and, finally, that she triumphs over the physiologist and philosopher, by the incomprehensibility of her works, and the demonstration of his own nothingness in the scale of her operations. We coincide with Dr. Lyall in the reasonableness of the following sentiments.

"We agree, therefore, in opinion with those who say, justice requires that when pregnancy has exceeded the ordinary or legal term, we ought not to presume the illegitimacy of the issue, unless other circumstances warrant this conclusion; but we think it preposterous to maintain, in the present state of knowledge, that our legislation ought to accommodate itself to the deviations from the ordinary period of pregnancy, by allowing more time than it does, at least by precedent, to establish the claim of legitimacy, and consequently the right of succession in such cases. Before any important changes be made, legislators will naturally demand more positive information than any we yet possess: upon that being acquired, it is probable that some modification of the laws respecting legitimacy and succession might become necessary."* xxiv.

In respect to the chief causes assigned for protracted pregnancy, we need say little. They have been considered as—aberrations of nature—hæmorrhages—mental emotions—and mechanical obstructions. These require no comment—and, therefore, we shall proceed at once to give some account of the individual evidence adduced in this celebrated cause.

1. *Mr. Charles Mansfield Clarke.* We shall avoid as much as possible the tautology of question and answer, and endeavour to give the sum and substance of the medical evidence in our own way.

Mr. C. considers 40 weeks the full period of a woman's gestation, under ordinary circumstances—and afterwards, on cross examination,—"forty weeks, I should say, is the extreme time." He never, in his life, knew any one instance of a labour having been retarded beyond the period just mentioned.

---

* The following extracts on legitimacy, from Beck's Medical Jurisprudence, may not be uninteresting in this place.

"Although the decisions on the subject of legitimacy have occasionally been very extraordinary and loose, yet considerable uniformity exists in the laws of various countries.

"The Roman law did not consider an infant *legitimate* which was born later than *ten months after the death of the father, or the dissolution of the marriage.* Such was also the French law prior to the revolution.

"A case is said to have been decided by a majority of judges of the Supreme Court of Friesland, by which a child was admitted to the succession, though not born till *three hundred and thirty-three days from the day of the husband's death,* which period wants only three days of *twelve lunar months.* The reader will find the details of this case, in Latin, by consulting Paris and Fonblanque's Medical Jurisprudence, vol. iii, p. 219.

"The Prussian civil code declares, that an infant, *born three hundred and two days after the death of the husband,* shall be considered *legitimate;* and a case has occured where one born three hundred and forty-three days after the death of the husband was adjudged a bastard by the *legislative commission* of that country.

"The civil code now in force in France contains the following provisions.

Mr. C. calculates the time from midway between the last menstruation and the one that would have succeeded, had not impregnation taken place. This is the gist of Mr. Clarke's evidence. It is founded entirely on his own personal experience; and is supposed to be rather too positive. Every man, however, who, like Mr. C. has had a very wide field for observation, is excusable for a certain degree of dogmatism on such occasions.

2. *Dr. Ralph Blegborough.* Estimates the period of human gestation at 39 weeks—"*but forty weeks I consider the ultimatum.*" This statement is founded entirely on his own personal experience of 34 years. Dr. Blegborough is equally as positive on this point as Mr. Clarke; for he observes that he does not think it *possible* a female can go beyond 40 weeks in pregnancy. There is a portion of Dr. Blegborough's evidence, however, which deserves further notice in this place. When asked—"are there any books in your profession which are reckoned works of authority at all?" He answers yes; but adds—"*There are very few men of very great eminence who have written books. Men who write books have seldom great practice: they are generally detailing the opinions of others, and not their own.*" A pretty compliment this to the luminaries of the profession, from Hippocrates downwards! Leaving the people of former days out of the question, is there a single eminent physician or surgeon in London, at this moment, who is not an author? And is Dr. Blegborough the only man of great practice in this metropolis? We consider the sentiment contained in the above passage as equally illiberal and unjust—to say nothing of its impolicy.

3. *Mr. R. R. Pennington.* Considers the usual period of utero-gestation as 40 weeks—never knew any one protracted beyond that more than three or four days—nor does he think it possible that pregnancy could be longer protracted—his reason for such conclusion is that—"from the effort of the uterus the woman and child would both die during the delivery." Mr. P. does not consider it possible that any agitation of mind, or any particular disorder could protract the gestation beyond the 40 weeks.

---

The child born in wedlock has the husband for its father. He may, however, disavow it if he can prove, that from the *three hundredth* to the *one hundred and eightieth day before its birth*, he was prevented, either by absence, or some physical impossibility, from cohabiting with his wife. An infant born before *one hundred and eighty days after marriage* cannot be disavowed by him in the following cases:—1. When he had a knowledge of his wife's pregnancy before marriage. 2. When he assisted at the act of birth, and signed a declaration of it. 3. When the infant is declared not capable of living. Lastly, the *legitimacy* of an infant born *three hundred days after the dissolution of marriage*, may be contested.

"It will be observed, that, by the last section, the child born after three hundred days is not positively declared a bastard, but *its legitimacy may be contested.* And Capuron, in remarking on this, observes, that it would probably be deemed legitimate, if no legal investigation should take place. The language of this law is also so put, that, in a contested case, all the learning of former times, and the innumerable cases related by medical jurists, might be brought forth to prove, that *eleven and twelve months* are *possible*, and even *probable*. I confess that I prefer the Scotch law, because it prevents this. It is concise and decisive. 'To fix bastardy on a child, the husband's absence must continue till within six lunar months of the birth. And a child born after the tenth lunar month, is accounted a bastard.'

The English law, on which our own (the American) is founded, does not prescribe a precise time. There are, however, some decisions which will show the ordinary course of adjudication."

4. *Dr. Robert Gooch.* Calculates nine calendar months—that is, for example, from the 25th March to the 25th December, as the period of utero-gestation. This is little more than 39 weeks. He thinks that 40 weeks exceed the usual term of pregnancy. Believes that it is sometimes a day or two more or a day or two less than this space of time. Thinks it quite impossible that pregnancy could be extended to ten calendar months. Dr. Gooch has been censured as casting, or, at least, propagating, an imputation on the veracity of medical men, on the authority of Dr. William Hunter. But we perceive that the imputation is directed to "men of science," and not to medical men in particular. "Dr. William Hunter himself said, there was no class of men who were more in the habit of recording unfaithfully than men of science; he said "they lie like the very devil." It is perhaps a pity that this quotation from Dr. Hunter should have been brought forward, but we cannot for a moment suppose that Dr. Gooch, who is a man of the most liberal sentiments, meant it to apply to medical men generally. There is no doubt that great error—much of it perhaps unintentional—has crept into medical records, and which gave occasion to the sarcastic remark of Cullen, that there were "more false facts than false theories in medicine;" but this, we think, by no means implies a stigma on the veracity of the medical profession.

4. *Dr. David Davis.* Would say that, as nearly as possible, the term of utero-gestation is nine calendar months—rather under than over this time.

5. *Dr. A. B. Granville.* We shall not advert to the long and minute examination which Dr. Granville underwent, respecting his own private history and medical education. We cannot see the reasons for this waste of time before the committee. Every man's evidence ought to rest on his own experience, without any very strict scrutiny where, how, and when, it was acquired. In respect to the question of pregnancy, Dr. Granville stated it as his opinion, that the ordinary period was embraced "between the 265th day subsequent to impregnation, and the 280th, or 40 weeks." The most important part, however, of Dr, Granville's evidence was the case of protracted utero-gestation occurring in the person of his own lady. Mrs. G. passed, that is, *missed,* her menstruation on the 7th April—and on the 15th August following, she quickened—that is, four months and six or seven days afterwards. In the early part of the first week in January, her confinement was expected, and a medical friend desired to hold himself in readiness to attend. Labour pains came on, but soon after subsided, and Mrs. G. went on till the 7th February, when labour returned, and delivery was speedy. The child was stronger than usual—was large—and was considered by Mrs. G. as well as by himself and Mr. A. T. Thomson, as a ten month child. Calculating conception then from the day before the *interruption,* (the latest that could be assigned) we should have a case *prima facie* of 306 days from impregnation to birth—but taking it at the middle period between the last menstruation and the interruption, we would have a case of 318 days.

Dr. G. knew a case of 285 days—of 290—of 300—and 315 days; but, in the last case, there was some doubt in his mind respecting the accuracy of the woman's statement.

6. *Dr. John Conquest.* Presumes the majority of cases are completed with the termination of the ninth calendar month—"but unquestionably, I have met with some cases which have far exceeded this date." Dr. C. took great pains in the investigation of two or three cases—"one woman was certainly pregnant *for, at least, ten months.*" One of these cases we shall quote.

"The case to which I refer is that of a woman who has borne six children. She is a woman possessing an unusual share of good common sense; and she engaged me to attend her during her second confinement before the period of quickening; she also engaged her nurse. She felt so confident that she should be confined at the anticipated time, that she had her nurse in her house; and it was not till the expiration of nearly *five weeks* from the time at which she expected to be confined that she was delivered, and delivered of a child of an unusual size. *At that time I disbelieved all the cases which I had previously heard; I had been in the habit of laughing at them as a public lecturer! but so strong was the evidence, from the most minute investigation of this case, that I was compelled to admit the accuracy of this woman's statement, and my former convictions were very much shaken.* The same thing occurred to this woman at her subsequent confinement: she exceeded the time then, certainly *four weeks;* she has since borne three children at the expiration of the ninth month; the three last children have been considerably smaller than the two intermediate children." 41.

In a note on Dr. Conquest's evidence, Dr. Lyall informs us that Dr. Hamilton, of Edinburgh, in his lectures, admits that, should a mother have an unexceptionable character, a decision ought to be given in her favour, though the child should not be produced till near *ten calendar months* after the absence or sudden death of the husband. In his own practice, however, he states, that he never knew a woman go beyond the eleventh menstrual period. Dr. C. underwent a long examination and cross-examination, but we do not deem it necessary to enter farther into the Doctor's depositions.

7. *Dr. Merriman.* Considered the "ordinary time of utero-gestation certainly about 40 weeks, or 280 days." In some cases he knew it to be extended to 285—in two or three instances, to 296—in one, to 303—and, in one, to 309 days. Upon the whole, he judged that the protracted gestation which was the object of investigation, was possible. For the particulars of some of these cases we must refer to Dr. Lyall's pamphlet.

8. *Dr. Hopkins.* Stated the ordinary period of pregnancy to be about 280 days. He knew this period exceeded "in one most positive case." When this most positive case, however, was sifted, we confess that it appears to us a very inconclusive one.

9. *Dr. Blundell.* Had personal experience of one case of utero-gestation being protracted beyond the ordinary time. The lady became pregnant on the 9th November, and she was delivered upon the 23d of August following. This was only 287 days; and there is no positive evidence to shew that the lady then exceeded the natural period by seven days; the case is inconclusive.

10. *Dr. John Power.* Believes, from what he has seen, that the period of utero-gestation may be protracted beyond the term of nine calendar months—that it may be extended to *eleven* calendar months, if not longer.

Several female accoucheurs were examined, but we do not see any necessity for introducing their evidence here. Our readers will perceive that, upon the litigated question of protracted utero-gestation, there was nearly an equal balance between the male part of the more eminent witnesses—to which part alone we would be inclined to attach any importance. Every one will be able to judge for himself respecting the part which he may feel disposed to take in this question. Without having any positive facts on which to ground our own opinion, but only the statements of females, and the conclusions of medical practitioners, we are inclined, as we said before, to admit the occasional, though very rare, aberration of Nature in this as in many other of her important operations. If she can bring forth a monster, with scarcely human shape or form, we see no reason why she should not occasionally accelerate or delay the usual evolution of the fœtus.

